# Stage-Specific Action of Matrix Metalloproteinases Influences Progressive Hereditary Kidney Disease

**DOI:** 10.1371/journal.pmed.0030100

**Published:** 2006-03-07

**Authors:** Michael Zeisberg, Mona Khurana, Velidi H Rao, Dominic Cosgrove, Jean-Philippe Rougier, Michelle C Werner, Charles F Shield, Zena Werb, Raghu Kalluri

**Affiliations:** 1Center for Matrix Biology, Department of Medicine, Beth Israel Deaconess Medical Center and Harvard Medical School, Boston, Massachusetts, United States of America; **2**Gene Expression Laboratory, Boys Town National Research Hospital, Omaha, Nebraska, United States of America; **3**Department of Anatomy, University of California San Francisco, San Francisco, California, United States of America; **4**Department of Surgery, University of Kansas School of Medicine, Wichita, Kansas, United States of America; **5**Department of Biological Chemistry and Molecular Pharmacology, Harvard Medical School, Boston, Massachusetts, United States of America; **6**Harvard–MIT Division of Health Sciences and Technology, Boston, Massachusetts, United States of America; **7**Division of Nephrology, Children's Hospital and Harvard Medical School, Boston, Massachusetts, United States of America; University of EdinburghUnited Kingdom

## Abstract

**Background:**

Glomerular basement membrane (GBM), a key component of the blood-filtration apparatus in the in the kidney, is formed through assembly of type IV collagen with laminins, nidogen, and sulfated proteoglycans. Mutations or deletions involving α3(IV), α4(IV), or α5(IV) chains of type IV collagen in the GBM have been identified as the cause for Alport syndrome in humans, a progressive hereditary kidney disease associated with deafness. The pathological mechanisms by which such mutations lead to eventual kidney failure are not completely understood.

**Methods and Findings:**

We showed that increased susceptibility of defective human Alport GBM to proteolytic degradation is mediated by three different matrix metalloproteinases (MMPs)—MMP-2, MMP-3, and MMP-9—which influence the progression of renal dysfunction in *α3(IV) ^−/−^* mice, a model for human Alport syndrome. Genetic ablation of either MMP-2 or MMP-9, or both MMP-2 and MMP-9, led to compensatory up-regulation of other MMPs in the kidney glomerulus. Pharmacological ablation of enzymatic activity associated with multiple GBM-degrading MMPs, before the onset of proteinuria or GBM structural defects in the *α3(IV) ^−/−^* mice, led to significant attenuation in disease progression associated with delayed proteinuria and marked extension in survival. In contrast, inhibition of MMPs after induction of proteinuria led to acceleration of disease associated with extensive interstitial fibrosis and early death of *α3(IV) ^−/−^* mice.

**Conclusions:**

These results suggest that preserving GBM/extracellular matrix integrity before the onset of proteinuria leads to significant disease protection, but if this window of opportunity is lost, MMP-inhibition at the later stages of Alport disease leads to accelerated glomerular and interstitial fibrosis. Our findings identify a crucial dual role for MMPs in the progression of Alport disease in *α3(IV) ^−/−^* mice, with an early pathogenic function and a later protective action. Hence, we propose possible use of MMP-inhibitors as disease-preventive drugs for patients with Alport syndrome with identified genetic defects, before the onset of proteinuria.

## Abstract 2

In a mouse model of Alport disease, matrix metalloproteinases appear to have a crucial dual role in disease progression, with an early pathogenic function and a later protective action.

## Introduction

Basement membranes (BMs) are dynamic structures which provide structural support and contribute to the acquisition of cellular phenotype and functional behavior [ 1, 2]. Major constituents of all BMs are predominantly laminins, nidogen/entactin, heparan sulfate proteoglycans, and type IV collagens—and the latter, as the most abundant BM-associated protein, also serves as a scaffold on which other BM proteins may interact [ 1– 3]. Type IV collagen includes six genetically distinct isoforms named α1(IV) to α6(IV) [ 1]. The six different isoforms are differentially expressed in various BMs and assembled into distinct networks, which potentially provide BM tissue–specificity [ 4]. While α1(IV) and α2(IV) chains are the most abundant isoforms in most BMs, distinct isoform compositions involving α3(IV)–α6(IV) are considered to represent specialized adaptation of BMs to site-specific requirements [ 1].

Mutations in type IV collagen have been linked to the genetic disorder Alport syndrome [ 5– 7]. Classically, Alport syndrome constitutes progressive renal disease associated with sensorineural deafness and occasional ocular defects [ 8, 9]. The renal disease associated with Alport syndrome causes hematuria, proteinuria, and progressive renal failure [ 9, 10]. The typical histopathological correlate of Alport disease in the kidney is splitting, thinning, and thickening of the glomerular basement membrane (GBM), which coincides with the onset of hematuria and proteinuria [ 11].

Several genetic studies have revealed that Alport syndrome is caused by mutations in the genes encoding for α3(IV), α4(IV), and α5(IV) chains of type IV collagen [ 9]. Mutations in the gene on chromosome X,q26–q48, which encodes for the COL4A5 chain, result in the X-linked form of Alport syndrome, accounting for approximately 85% of patients with Alport syndrome [ 7, 9,]. Mutations in the *COL4A3* or *COL4A4* genes, which encode for the α3(IV) and α4(IV) chains, cause autosomal recessive forms of this disease or, in rare occasions, autosomal dominant inherited forms of this disease [ 9, 12, 13].

Pathological mechanisms by which mutations in the *COL4A3*/ *COL4A4*/ *COL4A5* genes translate into renal disease are not fully understood [ 9]. The α3(IV), α4(IV), and α5(IV) chains of type IV collagen assemble into a unique network in the GBM, which is a central constituent of the filtration apparatus in the kidney. During kidney development, fetal α1(IV) and α2(IV) chain networks of the early GBM are replaced by the adult α3(IV), α4(IV), and α5(IV) chain networks in the mature GBM, and this isoform switching is arrested in patients with Alport syndrome, owing to defective assembly involving mutated type IV collagen genes [ 14]. In most patients with the X-linked form of Alport syndrome, the α3(IV), α4(IV), and α5(IV) chains are undetectable in the kidneys, suggesting that these three chains depend on each other for their incorporation into the GBM [ 9, 15].

As a major component of the ultra-filtration apparatus in the kidney, GBM, unlike any other BM, is constantly exposed to serum flow/pressure and thus needs to be functionally sound and needs to stringently maintain its structural integrity. It was previously proposed that α1(IV) and α2(IV) chain networks are less resistant to physical forces associated with constant filtration and exposure to endogenous local proteases when compared to more cross-linked α3(IV), α4(IV), and α5(IV) networks [ 9, 14]. Previous studies suggested that abnormal persistence of α1(IV) and α2(IV) isoforms in the Alport GBM is associated with an increased susceptibility to proteolysis by proteases [ 14]. However, studies utilizing mice that are deficient in both the α3(IV) chain of type IV collagen and matrix metalloproteinase (MMP)–9 failed to provide evidence for the role of MMP-9 in the initiation and progression of renal disease [ 16]. Here, we hypothesized that MMPs with overlapping substrate specificities could compensate for each other's absence in the setting of GBM-degradation, and we aimed to evaluate the impact of type IV collagen-degrading proteases on the progression of kidney disease associated with Alport syndrome using combined pharmacological inhibition of MMPs.

## Methods

### Mice

*MMP-2 ^−/−^, MMP-3 ^−/−^, MMP-9 ^−/−^,* and *α3(IV) ^−/−^* mice on a C57/BL6 background were generated as described previously [ 17– 20]. Double-mutant *(MMP-2 ^−/−^; MMP-9 ^−/−^)* mice were generated by crossbreeding *MMP-2 ^−/−^* and *MMP-9 ^−/−^* mice *.* Mouse studies followed the Institutional Animal Care and Use Guidelines. Age-matched mice were used for all studies.

### Human Samples

Nonfunctioning X-linked Alport syndrome (XAS) kidneys were obtained from two different male patients with XAS, in whom it was deemed appropriate to remove the diseased kidney at the time of transplant surgery. Kidneys from a 28-y-old patient with XAS (serum creatinine 459.7 μmol/l; patient XAS1) and a 42-y-old male patient with XAS (serum creatinine 1,582.4 μmol/l; patient XAS2) were used for immunostaining and immunoblot analysis for MMP expression. Normal human kidney tissue was obtained from a kidney that was not used for transplantation, and from histologically normal portions of resected kidneys with renal cell carcinoma. All specimen samples were provided by C. Shield III under appropriate patient consent and institutional approval.

### Preparation of Renal Basement Membranes

Renal basement membranes (RBMs) from mouse and human kidneys were isolated as described previously, with minor modifications [ 14, 21]. Mouse kidneys were obtained from 8-wk-old *α3(IV) ^−/−^* mice and from age-matched C57BL/6 control mice. Human RBM was obtained from nonfunctioning kidneys from male patients with confirmed XAS (from the patients described above) and from normal kidneys not used for transplantation [ 14, 22]. Kidney cortexes were shaved into an ice-cold solution of 0.85% NaCl containing protease-inhibitors (di-isopropylfluorophosphate [DFP], *N*-ethylmaleimide [NEM], -aminocaproate, and EDTA). The shavings were passed through a meat grinder and diluted with the above solution. The solution was suspended in 2% deoxycholate and stirred for 4 h at room temperature. The suspension was centrifuged, washed three times with ice-cold distilled water, and frozen at −20 °C immediately.

### Degradation of RBM by MMPs

RBM (500 μg) prepared from either control C57BL/6 mice, *α3(IV) ^−/−^* mice, normal human kidneys, or kidneys obtained from patients with XAS were suspended in 500 μl of 50 mM Tris-HCl, (pH 7.5), containing 1 mM calcium chloride and 0.02 % sodium azide as described previously [ 14, 21]. Recombinant active MMP-9, MMP-2, or MMP-3 (Calbiochem, San Diego, California, United States) was added at an enzyme-to-substrate ratio of 1:500, and digestion was continued at 37 °C. The reaction was stopped by addition of 50 μl of 150 mM EDTA, and the samples were frozen at −20 °C until analysis. The amount of type IV collagen degradation was quantified by measuring hydroxyproline released into the digestion supernatant as previously described [ 14, 21].

### Western Blot Analysis

Total protein was extracted from renal cortex using the T-PER Tissue Protein Extraction Reagent according to the manufacturer's recommendations (Pierce Biotechnology, Rockford, Illinois, United States). Protein concentration was assessed using the BCA protein assay (Pierce Biotechnology). For SDS PAGE, 200 μg of protein was loaded into each well. For Western blot analysis, proteins were transferred to Trans-Blot nitrocellulose membranes (Bio-Rad, Hercules, California, United States) and then probed with antibodies to MMP-2 (1:1,000, Calbiochem), MMP-3 (1:3000, Chemicon [ http://www.chemicon.com], MMP-9 (1:1000, Chemicon), and HRP-conjugated secondary antibodies for 1 h each at room temperature. After extensive washes, bands were visualized using reagents for enhanced chemiluminescence (ECL Western blotting kit, Amersham Biosciences, Little Chalfont, United Kingdom). For confirmation of equal loading, the blots were stripped and reprobed with antibodies to actin as described previously. The band corresponding to hexamers was cut out and protein was extracted.

### Immunohistochemistry

Immunohistochemical staining and electron microscopy was performed as previously described [ 23]. We used commercially available polyclonal antibodies to recognize MMP-2 (Calbiochem), MMP-3 (Chemicon), MMP-9 (Chemicon), and entactin/nidogen-1 (Sigma, St. Louis, Missouri, United States) in mice. For the detection of MMPs in human tissue sections, we used commercially available mouse monoclonal antibodies to MMP-2 (Calbiochem), MMP-3 (Calbiochem), MMP-9 (Calbiochem), and type IV collagen (Southern Biotechnologies, Birmingham, Alabama, United States). Briefly, 4-μm cryostat sections were fixed in 100% acetone at −20 °C for 10 min. The sections were incubated with primary antibodies at 4 °C overnight. Subsequently, the slides were washed three times in Tris-buffered saline (TBS) and incubated with donkey-derived secondary antibodies conjugated with Rhodamine or FITC (Jackson Immunoresearch, West Grove, Pennsylvania, United States). After four washes with TBS, the slides were mounted with Vectashield mounting media (Vector Laboratories, www.vectorlabs.com) and cover-slipped. The staining was analyzed using a fluorescence microscope (Zeiss Axioskop 2plus, http://www.zeiss.com) and visualized using Zeiss Axiovision digital imaging software. Areas of MMP-staining in the glomeruli and total cortex were quantified using NIH ImageJ software ( http://www.nih.gov). To assess glomerular staining, 20 glomeruli in randomly picked visual fields were assessed. To determine MMP-staining in the kidney cortex, visual fields at a magnification of ×200 were assessed in five mouse-kidney samples and ten human-kidney samples.

### In Situ Zymography

In situ zymography was performed as described previously [ 24, 25], but with some modifications. Briefly, the frozen blocks were sectioned sequentially using a cryostat microtome to prepare serial frozen thin sections (5 μm thickness). These thin sections were placed on a FIZ film (Fuji Photo Film, Tokyo, Japan). The films with sections were incubated for 12 h at 37 °C in a humidified chamber and stained with 1% Amido Black 10B in 40% methyl alcohol, 10% acetic acid, and 10% glycerol for10 min, destained in 40% methyl alcohol, 10% acetic acid, and 10% glycerol for 20 min, and kept in 20% glycerol for another 20 min. The sections were cover-slipped, sealed, and imaged using an Olympus BH-2 ( http://www.olympusmicro.com) microscope interfaced with a Spot-RT digital camera (Olympus). The gelatin in contact with the proteolytic areas of the section is digested, revealing white-to-pale blue areas on the otherwise black background. To confirm the specificity of in situ zymography, frozen sections were also placed on gelatin-coated films containing 1,10-phenanthroline (GI films), which is known to inhibit the activity of MMPs (unpublished data). In order to test the efficacy of the MMP-inhibitor cocktail, we employed the same MMP-inhibitors used for the in vivo studies at concentrations aimed to approximate sustained serum concentrations expected at the doses administered. MMP-2/MMP-9–inhibitor (2R)-[(4-biphenylsulfonyl)amino]- *N*-hydroxy-3-phenylpropionatamide and MMP-3–inhibitor *N*-isobutyl- *N*-(4-methoxylphenylsulfonyl)-glycylhydroxamic acid (Calbiochem) were used. The films were first immersed in the inhibitors (MMP-2/MMP-9–inhibitor at 800 nM and MMP-3–inhibitor at 300 nM), which were dissolved in saline (0.9% NaCl) at 300 μM for 10 min. The films were then thoroughly dried at room temperature and were used for inhibition studies [ 26].

### In Vivo Inhibition of MMPs

MMP-2, MMP-3, and MMP-9 were inhibited in *α3(IV) ^−/−^* mice starting after 4 wk and after 8 wk of age ( *n* = 6 mice in each group); control *α3(IV) ^−/−^* mice ( *n* = 6) received control buffer. Serum samples were obtained weekly, and urine samples were collected using metabolic cages once a week. MMP-2/MMP-9–inhibitor (2R)-[(4-biphenylsulfonyl)amino]- *N*-hydroxy-3-phenylpropionamide (Calbiochem) and MMP-3–inhibitor *N*-isobutyl- *N*-(4-methoxyphenylsulfonyl)-glycylhydroxamic acid (Calbiochem) were administered orally as described previously [ 27– 29]. MMP-2/MMP-9–inhibitor was suspended in vehicle buffer (saline including 0.4% Tween 80, 0.5% carboxymethylcellulose, and 0.9% benzyl alcohol) administered daily at a dose of 200 mg/kg po, which has been demonstrated to inhibit MMP-2 and MMP-9 in mice [ 28, 29]. MMP-3–inhibitor was administered at a dose of 75 μmol/kg dissolved in cornstarch [ 27]. Control mice were administered vehicle buffers alone.

### Statistical Analysis

All values are expressed as mean ± standard error of the mean unless specified. Analysis of variance (ANOVA) was used to determine statistical differences between groups using Sigma-Stat software (Jandel Scientific, San Rafael, California, United States). Further analysis was carried out using Students *t*-test with Bonferroni correction to identify significant differences. A level of *p* < 0.05 was considered statistically significant.

## Results

### MMP Localization during Progression of Renal Disease in *α3(IV) ^−/−^* Mice

The *α3(IV) ^−/−^* mice used in this study have a normal phenotype until 4–6 wk of age [ 30– 32]. Before 5 wk, the GBM contains minimal structural damage and the overall histology is healthy [ 33]. Additionally, it is important to stress that before week 5, no pathologic alterations in the tubulointerstitial compartment of the kidney can be detected [ 18, 31, 33]. After 4–5 wk post-partum, proteinuria and splitting of GBM is initiated [ 31, 33]. At 8 wk of age, severe splitting and thinning of the GBM are observed, associated with podocyte effacement and significant increase in urine protein levels, indicating malfunction of the glomerular filtration barrier [ 31, 33] At this stage, tubulointerstitial fibrosis, a common destructive pathway for many chronic nephropathies, is observed [ 30, 31].

**Figure 1:**
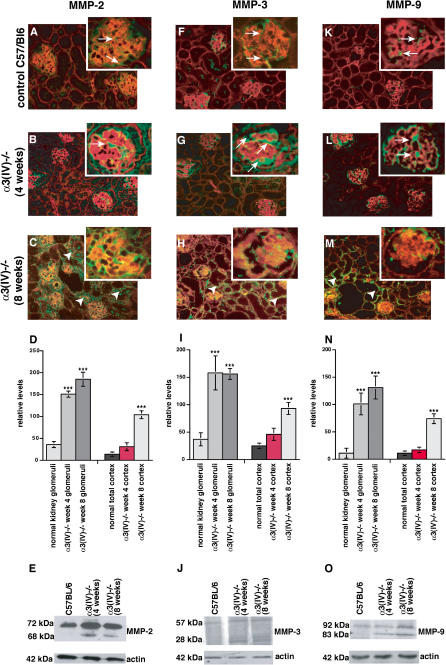
MMP Expression in Kidneys of *α3(IV) ^−/−^* Mutant and Wild-Type Mice Frozen sections of control C57/Bl6 mice and *α3(IV) ^−/−^* mice were stained with primary antibodies for MMP-2, MMP-3, and MMP-9 (FITC-green), and sections were counterstained with Rhodamine-labeled antibodies for entactin (red). (A) In control C57/Bl6 mice, MMP-2 is present sporadically in the glomeruli and in some proximal tubules (arrows). (B) After 4 wk of age, MMP-2 is increased in the glomeruli (arrows) in *α3(IV) ^−/−^* mice compared to age-matched control kidneys, even before manifestation of proteinuria. (C) After 8 wk of age (after manifestation of GBM-splitting and initiation of interstitial fibrosis), MMP-2 is increased in the interstitium (arrowheads) and in the glomeruli (arrows) in kidneys from *α3(IV) ^−/−^* mice. (D) MMP-2–staining was quantified using NIH ImageJ software in the glomeruli (left panels) and in total kidney cortex. Glomerular MMP-2–staining was significantly increased at 4 wk in *α3(IV) ^−/−^* as compared with control. *** *p* < 0.001 versus control. (E) MMP-2 expression was analyzed by immunoblot using total protein from the kidney cortex of single mice. A representative blot is shown, confirming increased expression of MMP-2 in *α3(IV) ^−/^* ^−^. The lower blot displays the control blot for actin to confirm equal loading. (F) Immunofluorescence staining revealed that MMP-3–staining is present in the glomeruli (arrows) of control C57/Bl6 mice. (G) MMP-3 is significantly increased in the glomeruli of *α3(IV) ^−/−^* mice after 4 wk of age. (H) In 8-wk-old *α3(IV) ^−/−^* mice, MMP-3 is up-regulated in the interstitium (arrowheads) and MMP-3 is also present in the glomeruli (arrows). (I) Quantification of MMP-3–staining revealed a significant increase of MMP-3–staining in the glomeruli and total cortex of *α3(IV) ^−/−^* mice at 4 wk and 8 wk of age. *** *p* < 0.001 versus control. (J) Immunoblot analysis revealed increased renal MMP-3 protein. (K) MMP-9 is present in the glomeruli of C57/Bl6 control mice (arrows). (L) The onset of proteinuria is preceded by a significant increase of MMP-9 in the glomeruli of *α3(IV) ^−/−^* mice (arrows). (M) MMP-9 is significantly up-regulated in the interstitium (arrowheads) and in the glomeruli (arrows) of 8-wk-old *α3(IV) ^−/−^* mice compared to control. (N) Quantification of immunofluorescence staining using ImageJ software confirmed a significant increase of MMP-9 expression in the glomeruli (left panels) and total cortex region (right panels) of *α3(IV) ^−/−^*mice as compared with control. *** *p* < 0.001 versus control. (O) Increased MMP-9 expression was confirmed by immunoblot.

We evaluated the expression of glomerular MMP-2, MMP-3, and MMP-9 in the context of glomerular diseases in the α3(IV) collagen-deficient mice. The analysis was performed at 4 wk of age when minimal splitting of GBM occurs (preceding podocyte effacement and proteinuria), and after 8 wk of age (when glomerular kidney disease is robust and progresses towards tubulointerstitial fibrosis). In normal wild-type mice, low baseline levels of MMP-2 were detected within the glomeruli, while it was absent within the tubulointerstitium ( Figure 1A). At week 4, before the onset of proteinuria in the *α3(IV) ^−/−^* mice, we detected significant glomerular expression of MMP-2, and this expression spread to the tubulointerstitial compartment when the mice exhibited proteinuria at 8 wk ( Figure 1B and 1C). Quantification of MMP-2–staining using NIH ImageJ software revealed a significant increase of glomerular MMP-2–staining at 4 wk of age in *α3(IV) ^−/−^* mice compared with control C57/BL6 mice ( Figure 1D). Increase of MMP-2–staining within the tubulointerstitial compartment was reflected by a significant increase in MMP-2–staining when the entire cortex region was analyzed ( Figure 1D). Presence of low levels of MMP-2 within the kidney cortex and increased MMP-2–expression in *α3(IV) ^−/−^* mice at 4 wk and 8 wk of age was confirmed by immunoblot using total protein lysates isolated from kidney cortex ( Figure 1E). Low levels of MMP-3 were present in the glomeruli of C57/BL6 control mice ( Figure 1F), but MMP-3 levels were significantly up-regulated in the week-4 *α3(IV) ^−/−^* glomeruli ( Figure 1F– 1I). At 8 wk in the *α3(IV) ^−/−^* mice, MMP-3 expression spread predominantly to the tubulointerstitial compartment ( Figure 1H– 1J). MMP-9 was observed predominantly in the tubules of normal mice ( Figure 1K); by 4 wk in the *α3(IV) ^−/−^* mice, low-level expression was observed in the glomeruli, which shifted into the interstitial space at 8 wk of age in the mutant mice ( Figure 1L– 1O). These results demonstrate that spreading of the disease from the glomeruli into the tubulointerstitial compartment is associated with MMP re-localization.

### Increased Renal Expression of MMP-2, MMP-3, and MMP-9 in the Kidney of Patients with XAS

**Figure 2:**
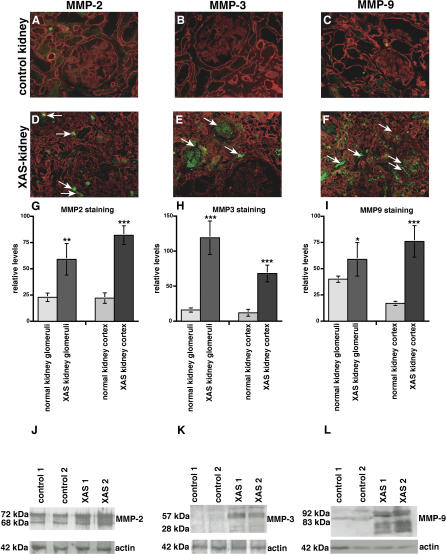
MMP Expression in Kidneys of Patients with XAS and in Normal Human Kidneys (A–F) Frozen sections of normal human kidneys and kidneys from patients with ESRD due to XAS were stained with antibodies to MMP-2, MMP-3, and MMP-9 (FITC-green), plus laminin (Rhodamine red). Representative stainings of samples obtained from a kidney designated for transplantation or from normal portions of resected kidneys with renal cell carcinoma are displayed. (A–C) Control. (D–F) Kidneys ( *n* = 2) obtained from male patients with XAS. (G–I) The bar graphs summarize the quantification of MMP-2–staining (G), MMP-3–staining (H), and MMP-9–staining (I). The left bars summarize the evaluation of MMP-staining in the glomeruli; the right bars summarize the MMP-staining in the total kidney cortex in each panel. *** *p* < 0.001 versus control; ** *p* < 0.005 versus control; * *p* < 0.01 versus control. (J–L) Total protein was isolated from normal human kidneys and from two kidneys obtained from two different patients with ESRD due to XAS. Protein was analyzed by SDS-PAGE and immunoblot using specific antibodies to MMP-2 (J), MMP-3 (K), and MMP-9 (L). The lower blots in each panel display the actin control blot to control for equal protein loading.

In order to assess the relevance of our findings of increased MMP expression in kidneys of *α3(IV) ^−/−^* mice with respect to human disease, we evaluated the expression of MMP-2, MMP-3, and MMP-9 in normal human kidneys and in kidneys obtained from patients with XAS. Whereas low levels of MMP-2, MMP-3, and MMP-9 could be detected by immufluorescence staining and immunoblotting in normal kidney tissue ( Figure 2A– 2C), MMP-2, MMP-3, and MMP-9 levels were significantly increased in kidneys obtained from patients with end-stage renal failure owing to XAS ( Figure 2D– 2L).

### Degradation of RBM by MMP-2, MMP-3, and MMP-9

**Figure 3:**
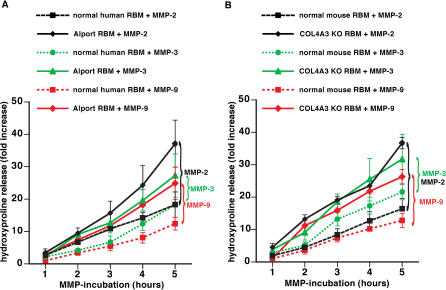
Increased Susceptibility to Proteolytic Degradation by MMPs of RBM from Patients with XAS and *α3(IV) ^−/−^* Mice (A) Degradation of human RBM by MMP-2, MMP-3, and MMP-9. RBM was isolated from human control kidneys and from patients with XAS. RBM was incubated with MMPs, and proteolysis was assessed by estimation of hydroxyproline release. The graph displays the relative increase of hydroxyproline over time. Experiments using normal RBM are represented by dotted lines; studies with RBM from patients with XAS are represented by a full line. Hydroxyproline release after MMP-2 incubation is displayed by black lines, MMP-3 incubation by green lines, and MMP-9 incubation by red lines. Proteolysis of Alport BM was significantly enhanced compared with normal human RBM by each of these MMPS. (B) Degradation of mouse RBM by MMP-2, MMP-3, and MMP-9. RBM was collected from normal C57BL/6 mice ( *n* = 6) and from α *3(IV) ^−/−^* mice ( *n* = 6) and subjected to active MMPs. Experiments using normal RBM are represented by dotted lines; studies with RBM from *α3(IV) ^−/−^* mice are represented by a full line. Hydroxyproline release after MMP-2 incubation is displayed by black lines, MMP-3 incubation by green lines, and MMP-9 incubation by red lines. Similarly to human Alport RBM, the RBM from *α3(IV) ^−/−^* mice displayed an increased susceptibility to proteolysis by MMPs.

MMP-2, MMP-3, and MMP-9—important BM-degrading enzymes—are found circulating in the blood of normal mice and humans [ 34– 36]. Here we investigated whether MMP-2, MMP-3, and MMP-9 were all capable of degrading human and mouse BM preparations (RBM, measured by hydroxyproline release as a function of time). MMP-dependent degradation was significantly enhanced when Alport RBMs or RBMs from α3(IV)-deficient mice were used ( Figure 3A and 3B). Without digestion, the hydroxyproline release did not increase over time, indicating that it was the type IV collagen composition, and not unspecific degradation of diseased GBM isolated from diseased kidneys, that accounted for this effect. These experiments suggest that MMPs present in the altered glomerular microenvironment are capable of degrading the Alport GBM much faster than the normal GBM.

### Compensatory Over-Expression of BM-Degrading MMPs in Mice Deficient in MMP-2, MMP-3, and MMP-9, or Deficient in MMP-2 and MMP-9 Only

Our studies point to the fact that enhanced degradation of GBM by BM-degrading MMPs could represent a crucial step in the renal disease progression observed in human Alport syndrome and also in the *α3(IV) ^−/−^* mice (see above). However, a recent study with mice deficient in both MMP-9 and α3(IV) collagen suggested that ablation of *MMP-9* does not alter renal disease progression in the *α3(IV) ^−/−^* mice [ 16]. We hypothesized that this observation could be due to compensatory over-expression of other BM-degrading MMPs when one of them is deleted. To test this hypothesis, we performed immunostaining experiments for BM-degrading MMPs in mice deficient in one or more MMPs.

**Figure 4:**
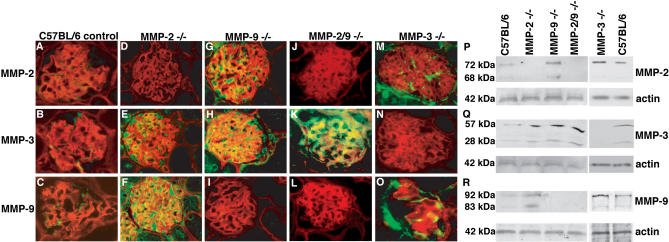
Compensation of BM-Degrading MMPs in *MMP-2 ^−/^* ^−^ *, MMP-3 ^−/^* ^−^ *,* and *MMP-9 ^−/−^* Mice, and in *MMP-2 ^−/−^* and *MMP-9 ^−/−^* Mice Immunohistochemical staining was performed with MMP-2, MMP-3, or MMP-9–antibodies (green) and with antibodies for entactin (red) on frozen kidney sections from *MMP-2 ^−/−^* and *MMP-9 ^−/−^* mice, and from *MMP-2 ^−/−^* and *MMP-9 ^−/−^* mice plus C57Bl/6 age-matched control mice. (A–C) Wild-type control kidney. Immunohistochemical staining demonstrated basal expression of MMP-2, MMP-3, or MMP-9 in the glomeruli of C57BL/6 mice. (D–F) *MMP-2 ^−/−^* kidney. In *MMP-2 ^−/−^* mice, the absence of MMP-2 was associated with a compensatory increase of MMP-3 and MMP-9. (G–I) *MMP-9 ^−/−^* kidney. MMP-9–deficiency was associated with increased MMP-2– and MMP-3–staining. (J–L) *MMP-2 ^−/−^* and *MMP-9 ^−/−^* kidney. The absence of both MMP-2 and MMP-9 was associated with a substantial increase of MMP-3 in the glomeruli of *MMP-2 ^−/−^* or *MMP-9 ^−/−^* double-mutant mice. (M–O) *MMP-3 ^−/−^* kidney. In *MMP-3 ^−/−^* mice, the absence of MMP-3 was associated with increased glomerular staining for MMP-2 and MMP-9. (P–R) Immunoblot analysis. Immunoblot analysis of total kidney-cortex protein preparations with antibodies specific for MMP-2 (M), MMP-3 (N), and MMP-9 (O) confirmed compensatory up-regulation of MMPs. The blots were stripped and reprobed with antibodies to actin in order to confirm equal loading (lower panels).

Mice that are deficient in MMP-2 *(MMP-2 ^−/−^),* MMP-3 *(MMP-3 ^−/−^),* and MMP-9 *(MMP-9 ^−/−^),* or which are deficient in MMP-2 and MMP-9 *(MMP-2 ^−/−^; MMP-9 ^−/−^)* only, are viable and do not display significant phenotypic abnormalities in the kidney [ 17, 19]. In the normal mice, MMP-2 and MMP-9, and to a lesser extent MMP-3, were detected in the glomeruli ( Figure 4A– 4C). In *MMP-2 ^−/−^* mice, compensatory up-regulation of MMP-3 and MMP-9 was observed in the glomeruli ( Figure 4D– 4F). In *MMP-9 ^−/−^* mice, the absence of basal MMP-9 expression was counterbalanced by a robust increase in MMP-2 and MMP-3 ( Figure 4G– 4I). In the double-mutant *(MMP-2 ^−/−^; MMP-9 ^−/−^)* mice, MMP-3 expression was dramatically up-regulated ( Figure 4J– 4L), whereas in *MMP-3 ^−/−^* mice, compensatory increase of MMP-2– and MMP-9–staining was documented as compared with C57BL/6 control mice ( Figure 4M– 4O). These findings were also confirmed by immunoblot analysis of total protein lysates isolated from the kidney cortex. Immunoblot analysis revealed compensatory up-regulation of other MMPs when MMP-2 and/or MMP-9 were deleted in mice ( Figure 4P– 4R). Such compensatory increase of MMPs with overlapping substrate-specificities has been demonstrated before in the uterus of matrilysin-deficient mice, where the absence of matrilysin was counterbalanced by increased MMP-3 expression, and in *MMP-3 ^−/−^,* mice in which the absence of MMP-3 (stromelysin-1) was associated with increased expression of MMP-10 (stromelysin-2) as well as of MMP-11 (stromelysin-3) [ 37]. These findings suggest that deletion of one MMP is not sufficient to halt the progression of renal disease associated with *α3(IV) ^−/−^* mice, owing to compensatory up-regulation of other BM-degrading MMPs.

### Combined Pharmacological Inhibition of MMP-2, MMP-3, and MMP-9 Activity in *α3(IV) ^−/−^* Mice

**Figure 5:**
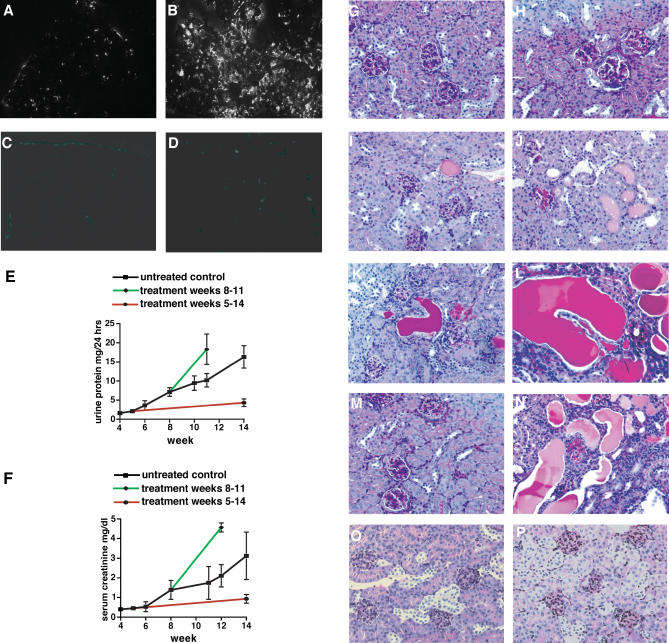
Effect of Combined MMP-Inhibition on the Progression of Renal Disease in *α3(IV) ^−/−^* Mice (A–D) In situ zymography. In situ zymography displayed gelatin-degrading activity in normal kidneys (A), which was substantially increased in kidneys of 14-wk-old *COL4A3 ^−/−^* mice (B). Incubation with the MMP-2, MMP-3, or MMP-9–inhibitor cocktail significantly blocked MMP activities in kidneys from wild-type mice (C) and kidneys from *COL4A3 ^−/−^* mice (D). (E and F) Renal function in *α3(IV) ^−/−^* mice. Urinary protein excretion (E) was measured in urine, which was collected over a 24-h period in metabolic cages. Serum creatinine (F) was determined in serum samples that were obtained bi-weekly from mice. The graphs display the progression of urinary protein excretion and serum creatinine levels from 4 to 14 wk of age. In each panel, disease progression is shown in untreated control mice (black), in mice that received MMP-inhibitors starting at 5 wk of age (red), and in mice that received MMP-inhibitors starting at 8 wk of age (green). (G–N) Kidney histology from *α3(IV) ^−/−^* mice. The images display representative periodic acid-Schiff (PAS)–stained kidney sections from *α3(IV) ^−/−^* mice, together with images displaying representative kidneys from untreated α *3(IV) ^−/−^* mice to illustrate disease progression (G–L). At the age of 3 wk, the kidneys appear completely normal (G). At 4 wk of age, a few glomeruli appear hypercellular, representative of inflammatory cells (H). At the age of 6 wk, a few tubules filled with protein casts appear (I). At 8 wk of age, a few sclerotic glomeruli are present, and atrophic tubules containing protein casts become more abundant (J). At the age of 10 wk, increased numbers of mononuclear cells associated with widening of the interstitial space indicate the onset of interstitial fibrosis (K). At 14 wk of age, the kidneys display severe tubular atrophy, glomerulosclerosis, and interstitial fibrosis, reflecting ESRD (L). A kidney section is displayed of a 14-wk-old *α3(IV) ^−/−^* mouse that received MMP-inhibitors from 4 wk of age (M). Initiation of treatment after week 4 (compare with H) led to substantially ameliorated disease after 14 wk (M, compare with L). A representative kidney section of a *α3(IV) ^−/−^* mice at week 11 that received MMP-2–inhibitors, MMP-9–inhibitors, and MMP-3–inhibitors, starting at week 8 and displaying enhanced progression of disease, is displayed (N, compare with K). (Original magnification of PAS-stained histologies: ×200). (O and P) Representative kidney sections from wild-type control mice at 4 wk of age (O) and 14 wk of age (P) are displayed. (Original magnification of PAS-stained histologies: ×200).

To evaluate the combined roles of MMP-2, MMP-3, and MMP-9 in the progression of renal disease in *α3(IV) ^−/−^* mice, we treated *α3(IV) ^−/−^* mice with a combination of MMP-inhibitors to block MMP-2, MMP-3, and MMP-9 at the same time [ 27, 28]. We used a combination of the MMP-2/MMP-9–inhibitor (2R)-[(4-biphenylsulfonyl)amino]- *N*-hydroxy-3-phenylpropionamide and the MMP-3–inhibitor *N*-isobutyl- *N*-(4-methoxyphenylsulfonyl)-glycylhydroxamic acid, which were administered orally as a combination-drug cocktail to normal mice, 5-wk-old *α3(IV) ^−/−^* mice, and 8-wk-old *α3(IV) ^−/−^* mice [ 29, 38]. We used in situ zymography to verify that MMP activity was increased in *α3(IV) ^−/−^* mice and that these MMPs were inhibited by the MMP-inhibitor cocktail ( Figure 5). Our findings do not exclude the possibility that additional MMPs with gelatin-degrading capacity were also inhibited, which would be a desired effect, as our goal was to evaluate the overall effect of BM-degrading MMPs in this disease model as opposed to delineating specific roles of single MMPs.

The progression of kidney disease in *α3(IV) ^−/−^* mice is consistent, resulting in end-stage renal failure after 14 wk of age. At the age of 3 and 4 wk, the mice present a normal kidney morphology ( Figure 5G and 5H). After 4–5 wk of age, disruption of the GBM results in proteinuria, which is reflected by tubules filled with protein casts after 6 wk of age ( Figure 5I). At the age of 8 wk, the kidney is marked by the onset of more severe tubulointerstitial disease, associated with tubular injury and interstitial infiltration with mononuclear cells ( Figure 5J). After 10 wk, the kidneys present with vast areas with interstitial inflammation ( Figure 5K), which results in severe fibrosis and end-stage renal failure after 14 wk ( Figure 5L). Initiation of MMP-inhibition, starting after 4 wk of age (before the onset of significant GBM damage and proteinuria) for 9 wk, dramatically delayed progression of renal disease in *α3(IV) ^−/−^* mice. At week 14, when end-stage renal disease (ESRD) was evident in untreated *α3(IV) ^−/−^* mice, the treated mice exhibited almost normal levels of serum creatinine and urine protein ( Figure 5E and 5F). We also observed significant improvement in renal histology compared to untreated mice ( Figure 5M, compare with Figure 5L). While the untreated Alport mice died at around 14 wk, the treated mice lived for an additional 5 wk, demonstrating significant extension of survival upon MMP-inhibition treatment.

In contrast, inhibition of MMPs starting at 8 wk of age, (after the onset of proteinuria and the emergence of interstitial fibrosis ( Figure 5J) for 4 wk, led to significant enhancement in disease progression with most mice dying between week 10 and week 12 ( Figure 5N, compare with Figure 5K). Serum creatinine levels and urine protein excretion was significantly enhanced after the start of MMP-inhibition, and renal disease rapidly progressed towards death by 11–12 wk of age, as compared with 14–15 wk in control untreated *α3(IV) ^−/−^* mice ( Figure 5E and 5F). This acceleration of disease was associated with significant increase in both glomerular and interstitial fibrosis.

### Ultrastructural Analysis of GBM in MMP-Inhibitor–Treated Mice

**Figure 6:**
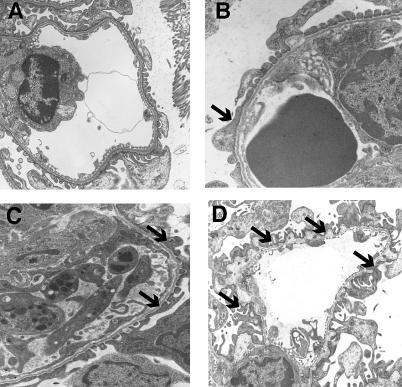
Ultra-Structural Analysis (Transmission Electron Microscopy) of GBM from Control and MMP-Inhibitor–Treated Mice (A) Control C57Bl/6 mice at 14 wk of age. The 5-wk-old normal mice also exhibited normal GBM architecture (unpublished data). (B) Kidneys from 5-wk-old *α3(IV) ^−/−^* mice before the onset of proteinuria, displaying the beginning of splitting of the GBM and podocyte effacement (arrow). (C). Kidneys from *α3(IV) ^−/−^* mice at 14 wk of age, which were treated from 5 wk of age with MMP-inhibitors for 9 wk, displayed moderate GBM-splitting and podocyte effacement (arrows). (D) Kidneys from 12-wk-old *α3(IV) ^−/−^* mice without treatment showed severe lesions of the GBM associated with podocyte effacement (arrows). Original magnification: ×12,250.

We used transmission electron microscopy to analyze the architecture of the GBM before and after treatment with MMP-inhibitors. Normal mice at 5 wk of age and 14 wk of age showed normal GBM structure (unpublished data; Figure 6A). Five-week-old *α3(IV) ^−/−^* mice treated for 9 wk with MMP-inhibitors exhibited significant protection of GBM architecture when compared to age-matched untreated mice with ESRD (14 wk) ( Figure 6B– 6D).

## Discussion

In the present study, we demonstrate that in *α3(IV)−/−* mice, a mouse model for human Alport syndrome, BMs and extracellular matrix (ECM)–degrading MMPs exhibit distinct functions in early and in late stages of disease progression. Additionally, we demonstrate the utility of MMP-inhibitors for significant attenuation of Alport renal disease, when administered at an early phase.

Interestingly, this discovery reminds us of recently published reports in the area of cancer research, where MMPs, in particular MMP-9, are identified as possessing an early pro-cancer property and a late anti-cancer activity [ 39, 40]. Therefore our results add to the growing body of literature that MMPs can exhibit context-dependent, contrasting functions. Thus it is vital to undertake a mechanistic evaluation of the means by which MMPs regulate disease progression before they can be considered as therapeutic targets.

The concept for the pathogenesis of Alport syndrome is that mutations in any of three chains of type IV collagen (α3, α4, and α5) lead to defective GBM. In the present study, we demonstrate that MMP-2, MMP-3, and MMP-9 are present in the glomeruli of normal adult mice at low, yet constant, physiological levels, confirming previous observations [ 41– 43]. However, the normal type IV collagen and laminin composition of the GBM appears sufficient to prevent accelerated deterioration of the glomerular filtration barrier despite the constant presence of MMPs. The possible importance of increased MMPs in the degradation of even normal GBM is demonstrated in studies in which increased levels of reactive oxygen species led to potentially enhanced activation of MMPs and deterioration of the glomerular filtration barrier [ 22, 44]. In this regard, pretreatment of rat GBM with H _2_O _2_ increases its susceptibility to degradation by proteases [ 44], and reactive oxygen species by themselves can increase the expression of MMPs in glomerular epithelial cells [ 45]. The importance of such exposure of the kidney GBM to MMPs and physical stress is further corroborated by the fact that in patients with XAS, the alveolar basement membrane in the lung—which normally contains the α3(IV), α4(IV), and α5(IV) chains of type IV collagen—remains normal in the functional sense [ 30].

In *α3(IV) ^−/−^* mice, the abnormal GBM composition is more susceptible to proteolytic degradation compared with wild-type mice, and the initial disruption of GBM is associated with further increased MMP levels from infiltrating monocytes, further augmenting GBM-degradation in these mice [ 25]. Combined inhibition of BM-degrading MMPs significantly delayed the onset of proteinuria in these mice when they were treated before proteinuria was observed. Similarly, we demonstrated that RBM isolated from human kidney samples with XAS displays an increased susceptibility to proteolytic degradation by MMP-2, MMP-3, and MMP-9, and that the progression of renal disease in patients with XAS is associated with increased expression of MMP-2, MMP-3, and MMP-9 as in the *α3(IV) ^−/−^* mice. These results suggest that MMP-mediated degradation of GBM is a key event in the progression of renal disease in *α3(IV) ^−/−^* mice and in patients with XAS. Such MMP up-regulation is not unique to Alport disease only. However, in the context of Alport disease, such MMP up-regulation degrades the GBM faster owing to defective type IV collagen composition, whereas the degradation kinetics are unaltered in other kidney diseases. While our results suggest that MMP-2, MMP-3, and MMP-9 are the principal MMPs involved in this process, we cannot exclude the possibility that other BM-degrading MMPs are also likely involved. In contrast, after the onset of proteinuria and accumulation of significant damage to the GBM, the renal disease in these mice is no longer dependent on MMP activity and, interestingly, inhibition of BM-degrading MMPs at this stage leads to significant acceleration of renal disease. Collectively, these results suggest that, before the onset of proteinuria, MMPs are responsible for disease progression, but after induction of proteinuria and the emergence of fibrosis, MMPs protect against rapid acceleration of disease.

Tubulointerstitial fibrosis is considered the most common destructive pathway associated with chronic nephropathies, including Alport syndrome [ 9, 46]. Renal fibrosis is characterized by a remodeling of the interstitial ECM, resulting in an excessive deposition of ECM (mainly collagens type I, type III, and type IV), which culminates in significant deterioration of renal excretory function [ 9, 47]. The notion that enhanced degradation of ECM is essential for preventing the accumulation of ECM during fibrosis is not new, and many investigators have reported on this concept [ 46]. In such studies, MMPs are generally considered to be reno-protective, owing to their potential to cleave interstitial ECM constituents and reduce scarring [ 47]. Decreased expression of MMP-2 and MMP-9, associated with increased expression of their inhibitors—TIMP-1, TIMP-2, and TIMP-3—was demonstrated in various independent animal models of renal fibrosis and in human biopsies [ 47– 50]. Several studies using animal models of fibrosis demonstrated that a pharmacologic increase of MMPs can inhibit the deposition of ECM within the interstitium [ 51– 53]. These observations are consistent with our findings in the context of renal fibrosis. We propose a new model for the role of MMPs in the progression of renal disease associated with Alport syndrome. Before the onset of proteinuria and significant GBM damage, enhanced MMP activity is responsible for disease induction owing to the capacity of MMPs to degrade Alport GBM more rapidly. After the onset of proteinuria, however, the MMPs are beneficial as they are responsible for removal of ECM scarring associated with fibrosis.

Finally, our results offer the possibility of using MMP-inhibitors as preventive drugs for patients with Alport syndrome with an established mutation/deletion identification and correlative family history. In this regard, MMP-inhibitors are currently in late-stage clinical trials for many other indications, and hence are suitable for potential testing in this setting. However, we also provide evidence that interference with the MMP system in the context of a chronic kidney disease can be a double-edged sword that needs to be approached with great care. Up-regulation of MMPs to inhibit, or even reverse, the progression of fibrosis has been proposed as a potential therapeutic approach. Our findings now demonstrate that MMPs can be equally involved in the initiation of disease. Collectively, our findings suggest that, while the inhibition of MMPs before the onset of kidney disease in patients with Alport syndrome could be beneficial, this inhibition would have an opposite effect once the disease process is initiated.

**Patient Summary**

**Background**

Alport syndrome is an inherited disorder in which affected patients get progressive renal disease associated with nerve deafness and, occasionally, eye problems. The disease is caused by mutations in the genes that make up one type of collagen—type IV. This substance is an essential part of the structure of several organs in the body, including the glomerular basement membrane which lines the part of the kidney involved in filtering toxins from the blood into the urine. The abnormalities in the collagen cause the glomerular basement membrane to split, thin, and thicken irregularly. These changes lead to blood and protein appearing in the urine and, eventually, to renal failure.

**Why Was This Study Done?**

Previous work has suggested that the glomerular basement membrane in patients with Alport syndrome is unusually susceptible to being damaged by certain enzymes The researchers wanted to investigate this idea further in a mouse that has a disease very similar to that of humans, and also in samples taken from kidneys of people with Alport syndrome.

**What Did the Researchers Do and Find?**

They found that the glomerular basement membrane in people with Alport syndrome was very susceptible to being broken down by three different enzymes, known as matrix metalloproteinases (MMPs) If the mice with disease similar to Alport syndrome were treated before the onset of kidney disease, with drugs that prevented these enzymes from working, they had a less rapid onset of kidney disease. However, giving the drugs after the kidney disease had begun, led to the disease becoming worse in the long run, and in the early death of the mice.

**What Do These Findings Mean?**

This study in mice and human kidney samples shows that MMPs are important at two stages of the kidney disease of patients with Alport disease. If MMPs are inhibited early on, it might be possible to slow the progression of the disease. However, once the disease is established, inhibiting the MMPs would make things worse. Much more work will need to be done to see whether there are drugs that inhibit MMPs that can be given safely to patients and, if so, when exactly the best time is to give them to humans.

**Where Can I Get More Information Online?**

Here are listed several Web sites with information on Alport syndrome.

Medline Plus:

http://www.nlm.nih.gov/medlineplus/ency/article/000504.htm

Genetics Home Reference, from the US National Library of Medicine (particularly on the genetics of Alport syndrome):

http://ghr.nlm.nih.gov/condition=alportsyndrome

National Kidney Foundation:

http://www.kidney.org/kidneydisease

## Abbreviations

ANOVA: analysis of variance
BM: basement membrane
DFP: di-isopropylfluorophosphate
ECM: extracellular matrix
ESRD: end-stage renal disease
GBM: glomerular basement membrane
MMP: matrix metalloproteinase
NEM: *N*-ethylmaleimide
NIH: National Institutes of Health
PAS: periodic acid-Schiff
RBM: renal basement membrane
TBS: Tris-buffered saline
XAS: X-linked Alport syndrome
